# Mexican validation of the International Affective Digitized Sounds second edition (IADS-2) and additional sounds

**DOI:** 10.1038/s41598-022-26320-w

**Published:** 2022-12-17

**Authors:** Norberto E. Naal-Ruiz, Luz M. Alonso-Valerdi, David I. Ibarra-Zarate, Alba Serrano-Cena, Gustavo Navas-Reascos

**Affiliations:** 1 Escuela de Ingenieria y Ciencias, Tecnologico de Monterrey, 64849 Monterrey, Nuevo Leon Mexico; 2 Escuela de Medicina y Ciencias de la Salud, Tecnologico de Monterrey, 64849 Monterrey, Nuevo Leon Mexico

**Keywords:** Human behaviour, Limbic system, Acoustics, Emotion

## Abstract

Affective stimuli have been extensively used in emotion research for a better understanding of emotion regulation. Sound ratings, specifically non-verbal sounds, are biased by demographic indicators such as sex and nationality. Therefore, it is crucial to characterize sounds prior to their use in emotion research. This study aims to validate the IADS-2 database and additional sounds in a sample from the Mexican population. Three hundred twenty-nine participants born and raised in Mexico remotely listened to 174 sounds in monophonic format. They rated sounds according to the valence-arousal-dominance model using the Self-Assessment Manikin test. Results positively correlated to those of previous studies. Sex differences were observed only in dominance between female and male groups, contrary to the results from Portuguese, American and Japanese validations. Geographic region analysis demonstrated differences in arousal, indicating the need for additional research on occident and south regions. Furthermore, when conducting affective research, headphones and audio quality should be considered, primarily to reduce variability due to audio-related aspects, and to avoid changes in emotional experience. Finally, this study supports the feasibility of remote affective sound experiments over the internet as reported in previous research.

## Introduction

The study of emotions has been a compelling research topic in the last decade, primarily to regulate human behavior. The most recurrent models for the study of emotions have been the following two: (1) the continuous dimensional model of valence and arousal^1–3^, and (2) the discrete model in the form of categories (e.g., anger, disgust, fear, sadness, happiness, surprise)^4–7^. In the continuous model, valence measures activations of two systems, one preventing dangerous situations (aversive system), and the other promoting well-being (appetitive system)^8^. Valence is categorized between pleasantness and unpleasantness, or positive and negative emotions. Arousal measures activation levels of stimuli between calmness and excitation. According to Plutchik’s model^9,10^ discrete emotions can be divided in terms of valence and arousal, grouping emotions with similarities in more complex quadrants. An additional dimension used in the study of emotions is dominance, which measures the level of perceived control of emotions after stimulation^8^. The three dimensions constitute the valence-arousal-dominance (VAD) model^11^. The VAD model has been employed in psychometric tests as the Self-Assessment Manikin test (SAM), representing opposite sides of dimensions in-9 point scales^12–15^, one for each emotional dimension and assisted by graphic representations. For valence, the lowest point is represented by a frowning figure, and the highest by a smiling face. Low arousal is depicted by a figure with eyes closed, and high arousal by a wide-eyed figure. Low dominance is represented by a manikin small in size, and high dominance by a manikin larger than the bordered space of the figure^12^. This test has been employed in validations of affective stimuli since the graphics are regarded as cultural-free^16^. Still, it is difficult to differentiate a specific emotion unless additional questionnaires evaluating a determined emotional condition are added, and demographic indicators such as sex and nationality are taken into account^17^.

According to Schafer^18^, elements in a soundscape can cause positive or negative emotional responses related to geography, customs, and traditions of a community. In his work, sounds that contribute to shaping human behavior are divided into Keynotes, Signals, and Soundmarks. Keynotes refer to sounds present in the environment and are not consciously perceived or listened to, such as birds at a park. Signals are those auditory messages that transmit a message and are consciously listened to, such as the siren of an ambulance. Soundmarks are geographical representative sounds that are understood and cared for by a community such as a volcanic eruption sound, identified by communities around it. In addition to the previous sounds, music has been considered as enduring evidence of customs and feelings shaped by society^18^. Other sounds such as pure tones, white and pink noise have been widely used for therapeutic purposes, like tinnitus relief^19^ and analgesic stimuli for pain sufferers^20^.

There is evidence showing that auditory information from an environment shapes emotions and human behavior of listeners^21^. To assume that sounds are perceived worldwide with a defined emotion could bias results in research. Hence, it is necessary to characterize sound preferences in communities before their application in emotion research. To reduce cultural and demographic influences, non-verbal sounds such as those from the International Affective Digitized Sounds (IADS-2) database^22^ have been employed in the study of emotions since samples from different countries have shown to have similar ratings^17,23^. Currently, the IADS-2 database has been validated in samples of the American^22^, Portuguese^17^, and Japanese^23^ populations. The present study aimed to validate the IADS-2 database and additional sounds in a Mexican sample for emotion research using the VAD model. The study was conducted in an online form, that is, by scheduling virtual meetings and streaming audio files in real time. Emotional ratings were analyzed in four categories: (1) samples from United States (USA)^22^, Portugal (POR)^17^, Mexico (MEX, current study), and Japan (JPN)^23^; (2) sex groups, (3) Mexican geographic regions; and (4) type of headphones. Three countries were considered for the country analysis since those were available at the moment of the study. Results from the Spanish validation were excluded since the first database of IADS was used, having less sounds than IADS-2^8,24^. Sex groups are also analyzed to compare ratings to those of previous validations. Due to the large population in Mexico and its broad cultural variety, geographic region analysis is included in this work. Additionally, headphone analysis in three general models is also taken into account to measure the effect of the playback system.

## Methods

### Sample

Three hundred twenty-nine individuals participated in the study and rated 174 sounds, 167 from IADS-2 and seven additional sounds. The age of participants ranged between 17 and 60 years old (M = 24.467, SD = 7.133, 143 female, 186 male). From the sample, 88.15% of participants reported to be right-handed, 6.38% of them were left-handed, and 5.47% of them were ambidextrous. Regarding audio expertise, 33.43% of participants reported to have audio experience, that is, to work or study in music- or audio-related fields. On the other hand, 66.57% of them reported to have none. Most participants were undergraduate students or individuals who recently finished an undergraduate program (82.07%). The rest of them were high school students (2.43%), graduate students (15.50%), or individuals who recently finished previous studies in those categories. Most students were from Tecnologico de Monterrey. Previous research has reported that emotional ratings from university samples are valid as referential points for research^25^. Tecnologico de Monterrey is one of the top universities in Mexico according to the QS Ranking^26^ with students from many states and with facilities all over the country. Then, participants from all Mexican regions were included in the study (north = 35%, occident = 12%, orient = 11%, south = 4%, center = 38%). The distribution of participants in Mexican regions according to the 2020 National Institute of Statistics, Geography and Informatics (INEGI) Census^27^ is shown in Fig. S1 in the supplementary information section. It can be observed that the center and orient regions have almost the same size in both graphs. Nevertheless, the north region had a greater number of participants compared to the south and occident. From the sample, 40.12% of participants used circum-aural (around the ear), 43.47% of them used intra-aural (inserted in the ear), and 16.41% of them used supra-aural (over the ear) headphones.

Previous research has shown that approximately 100 ratings per sound are needed to observe the “boomerang” shape in the bi-dimensional space of valence and arousal^22^. Nevertheless, it has been reported that participants rating 100 sounds present fatigue^17^. Therefore, participants were randomly divided into three groups of 100 individuals each. Each group rated 58 sounds, which were also randomly assigned to the three groups at the beginning of the experiment. In this form, each subject rated just once each of the 58 sounds. At the end of the experiment, each group had the following participants: (a) Group 1 = 112; (b) Group 2 = 109; and (c) Group 3 = 108. For country comparison, results from previous validations in USA (at least 100 ratings per sound, approximately half were female participants. This information is reported as shown in the technical report B-3^22^), POR^17^ (N = 300, 254 females, 46 males) and JPN^23^ (N = 207, 104 females, 103 males) were included. These were the datasets available at the time of the analysis of this experiment.

### Materials

All auditory stimuli (167) from IADS-2 were used. Sounds were not uploaded nor stored in cloud-based platforms, but streamed once in real time through the internet. Audio files were in monophonic format at 44.1 kHz sample rate with 16-bit depth. In addition to the previous sounds, two noise files (pink and white noise) generated with a Matlab algorithm^28^ and 6-s song excerpts for five music genres were included (fragments in minutes taken from songs are expressed between parentheses):

- Pop: Bad Habits by Ed Sheeran (from 2:37 till 2:43)
- Banda: Ya te la Sabes by Julion Alvarez y su Norteño Banda (from 0:03 till 0:09)
- Electronic: Rasputin by Majestic and Boney M (from 0:07 till 0:13)
- Cumbia: Como te voy a olvidar by Los Angeles Azules (from 0:06 till 0:12)
- Reggaeton: Que Mas Pues? by J Balvin & Maria Becerra (from 3:27 till 3:33).

Therefore, seven more sounds were added to the 167, giving a total of 174 sounds. A survey conducted in 2017 showed that the preferred music genres in Mexico were Pop, Rock, Banda music, Electronic music, and Cumbia^29^. Reggaeton was a popular genre among young people. Music excerpts were searched in the top Mexican charts of streaming services (i.e., Spotify) and radio charts (i.e. Billboard). Then, songs were bought from the iTunes Store (Apple Inc.) if they had at least 6-s instrumental passages. Cumbia did not have a song in tendency during the period of the study. Nevertheless, one of the most popular cumbia songs found in several streaming services playlists was chosen. IADS-2 already had a rock music excerpt and then, an additional rock song was not included. Sound presentation order was randomized every session to discard an effect due to the train of sounds. The additional sound files were matched in length, digital levels, and number of channels (mono files) to those from the IADS-2. Due to COVID-19 pandemic limitations and the large sample of participants, sound pressure level and frequency-related parameters could not be controlled since it was a remote experiment. For this reason, a 1 kHz file was created at − 20.56 dBFS RMS level, which is the median average dBFS RMS level from all audio files reported in the first IADS-2 validation^12^. The median was selected because the RMS values of all sounds did not show evidence of a normal distribution on the Ryan-Joiner normality test (rj = 0.948, *p* < 0.01), and the mean value would have been less accurate. This tone served for computer digital audio calibration purposes. The application Zoom (Zoom Video Communications, Inc.) was used for the virtual meeting, and the VST3 Listento audio plug-in (Audiomovers) was used to play the sounds in high quality. Audio files were played in REAPER digital audio workstation (Cockos Incorporated) on an Apple Macbook Pro computer (Apple, Inc.). The Listento transmitter VST3 plug-in was inserted in the master track of the audio project. Plug-in parameters were PCM 16-bit audio quality with latency varying between 0.5 and 2 s, depending on the internet speed. A registration form, informed consent and an online SAM test was given to the participants through the Google Forms (Google, LLC) platform.

### Experimental setup

This study was approved by and conducted in accordance with guidelines of the ethics committee of the School of Medicine from Tecnologico de Monterrey (CONBIOETICA-19-CEI-011-20161017). Informed consent was obtained from all participants before experimentation. Before the virtual meeting, participants were recruited by a national public announcement posted through social networks. The post showed a registration link. Information such as age, sex, nationality, state, handedness, education level, audio expertise, auditory acuity, neurological health and time availability was asked in the registration form. Due to COVID-19 pandemic limitations and the large sample, a personalized hearing and neurological assessment were not planned. Instead, an informed consent, questions related to auditory acuity and neurological health were presented in the registration link. Hearing questions were proposed by an otolaryngologist, and the neurological questions were about neurological condition suffering.

Participants fulfilling inclusion criteria (age 17 years old and above, born and living in Mexico, auditory and neurological health) were then asked to attend a scheduled virtual meeting through a Zoom link. Meetings were scheduled according to participants availability reported in the registration form. Participants with the same availability assisted to the same meeting. Therefore, several virtual meetings were performed until completing 100 ratings per sound. Every participant was requested to use headphones during the experiment and attend to the meeting on a computer. Upon arrival, the name of each participant was verified. After all participants had arrived at the virtual meeting, a Google Forms survey and a Listento link was sent to the participants through the application chat. Afterward, a 1 kHz calibration file was played through Audiomovers, and participants were asked to adjust the computer volume to a pleasant level without being too soft. Once the calibration procedure finished, participants were instructed not to change the volume level while rating sounds and the experiment began with the guidelines provided with the IADS-2.

**Figure 1:**
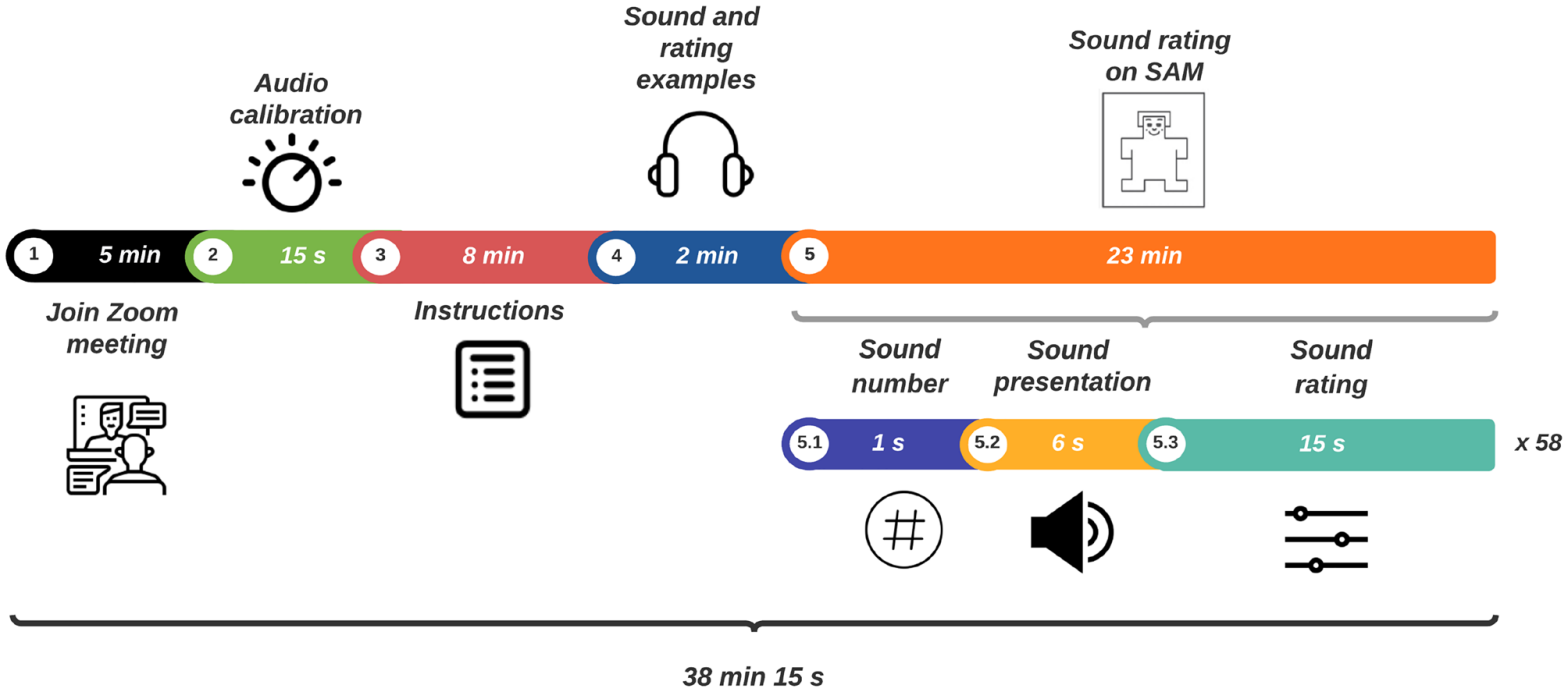
Experimental design: from joining virtual meeting to SAM. Participants joined a Zoom online meeting.

**Figure 2:**
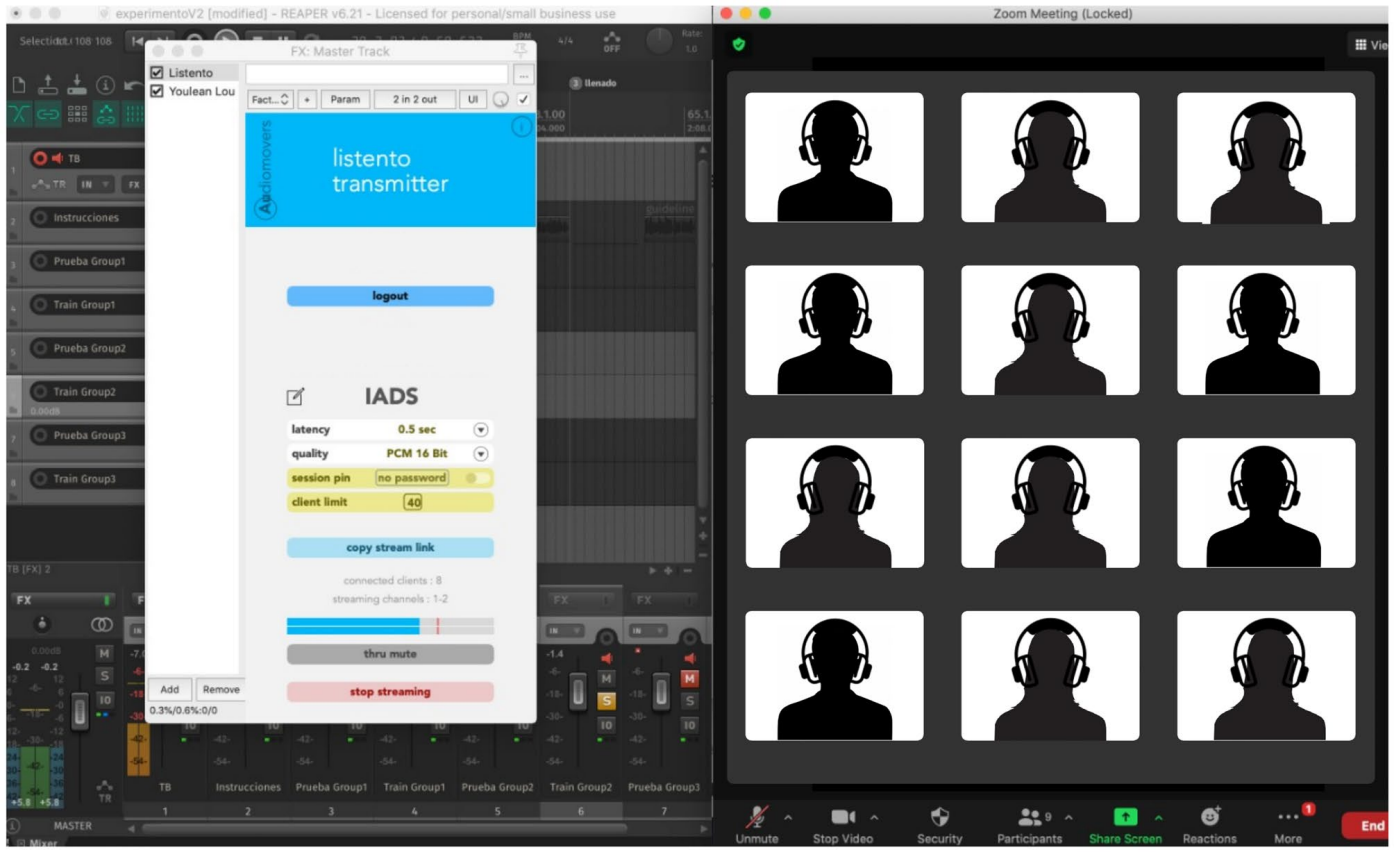
Virtual meeting. High-quality audio was transmitted in real time over the internet. Participants wore headphones during the experiment.

At the beginning of the meeting questionnaire, participants were requested to report their headphone models and select a headphone type: intra-, supra- or circum-aural. Finally, participants were asked to fill in SAM in terms of valence, arousal and dominance. For each sound, participants could tick a checkbox if they heard audio errors or if they did not listen to a sound. This strategy prevented to include ratings from those sounds in statistical analysis. Data from participants with many missed or audio error ratings were discarded. In every session, an additional researcher was remotely connected to the experiment for supervising audio playback. Figure 1 illustrates the experimental procedure and Fig. 2 shows an example of a virtual meeting.

### Statistical analysis

Ratings from participants were averaged, excluding missed and audio error ratings. Handedness, education level and audio expertise were not included as categories in statistical analysis since a larger part of participants were right-handed, students and did not have audio expertise. Descriptive statistics are given for all sounds and categories. Additional sounds were analyzed separately from IADS-2 since previous validations do not contain these particular excerpts. Statistical testing was conducted in Minitab 21.1. Pearson’s correlation test was performed on the mean emotional ratings of the IADS-2 sounds between categories. Linearity between variables was confirmed before choosing the test by means of scatter plots. To observe significant differences within categories and the mean ratings of the VAD model, multivariate analysis of variance (MANOVA) was employed for each category individually. Mean ratings of valence, arousal and dominance were the response variables, and the groups in categories for countries (USA, POR, MEX, and JPN), sex groups (male and female), Mexican regions (north, occident, orient, south and center), and type of headphones (intra-aural, supra-aural and circum-aural) were the independent factors. If significance was observed, a two-sided multiple comparison using Bonferroni’s method was used to compare groups within categories. Level of significance was established at 0.05.

**Figure 3:**
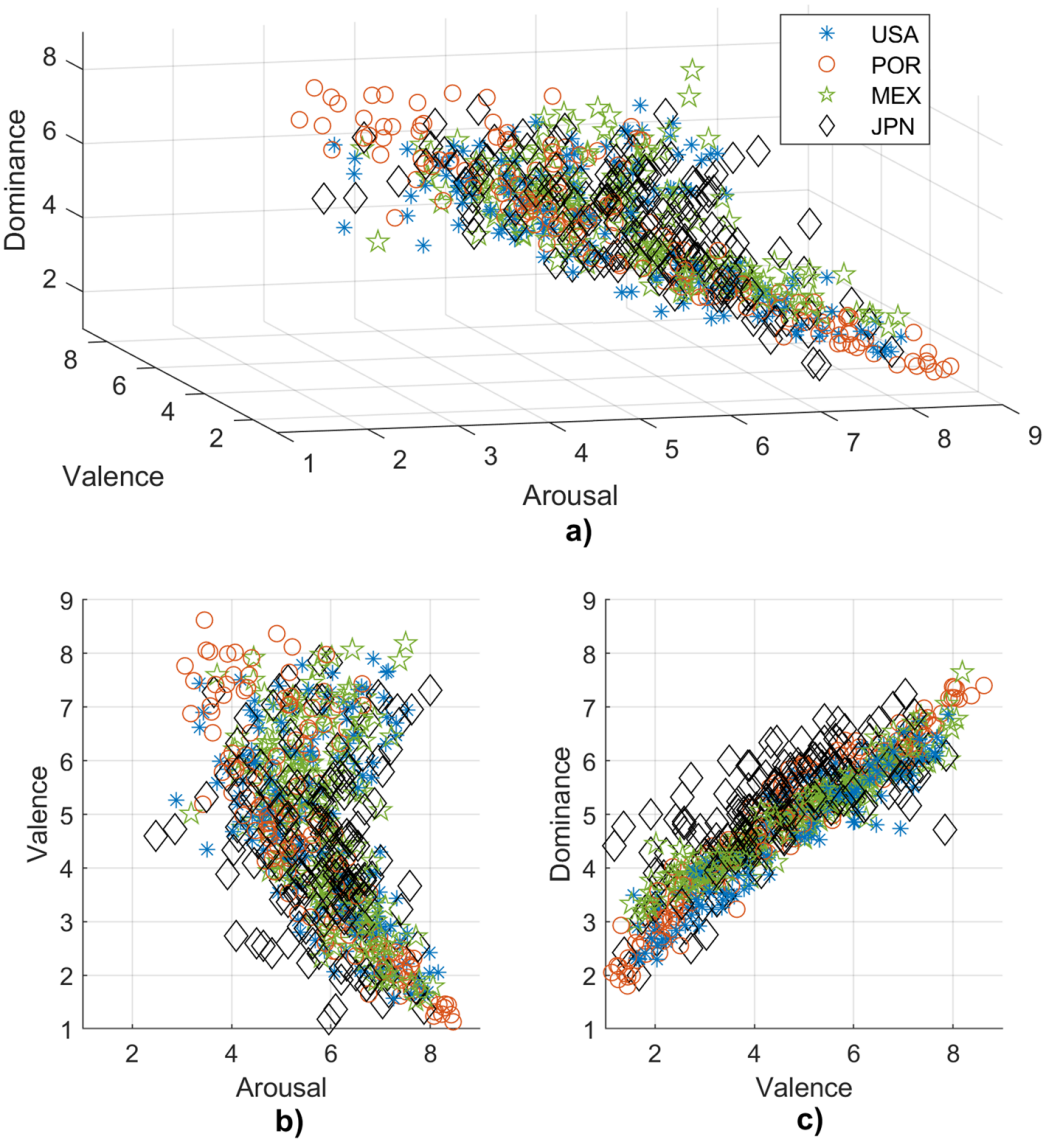
Distribution of mean ratings in the VAD model. The marker type indicates the mean ratings from SAM of different countries for the 167 IADS-2 sounds (167). For MEX, the 329 ratings were considered to calculate the mean rating of each sound. (**a**) Three-dimensional space. (**b**) Arousal-valence plane. (**c**) Valence-dominance plane.

Lastly, a regression analysis was computed to model the relationship between valence and dominance, being the dimensions with greatest associations as observed in Fig. 3c. Valence ratings from all countries were chosen as the input data and dominance ratings as the predictors. Analysis was performed using the regression learner app in Matlab, choosing a 20% validation holdout due to the large dataset (668 ratings including JPN, 501 excluding JPN).

## Results

### Countries

Figure 3 presents the mean sound ratings from previous research, including the current study: USA^22^, POR^17^, MEX and JPN^23^. Figure 3a presents the ratings on the three-dimensional space of the VAD model. For MEX, the 329 ratings were used to calculate the mean rating of each sound. As observed in Fig. 3b, data from most samples have the same behaviour, showing a “boomerang” shaped curve as reported in previous research^17,22^. It is important to note that aversive sounds seem to have high arousal ratings, compared to pleasant sounds being widespread in the arousal dimension.

**Table 1:** Descriptive statistics for each dimension and country.

		Val				Aro				Dom			
		M	SD	Min	Max	M	SD	Min	Max	M	SD	Min	Max
**Country**	USA	4.784	1.756	1.570	7.900	5.843	1.157	2.880	8.160	4.714	1.166	2.290	6.860
	POR	4.549	2.041	1.130	8.622	5.711	1.323	3.061	8.460	4.815	1.496	1.800	7.402
	MEX	4.875	1.714	1.524	8.187	5.864	0.932	3.187	8.056	4.970	0.940	2.991	7.645
	JPN	4.397	1.544	1.182	7.864	5.848	0.996	2.480	8.000	5.044	1.044	2.000	7.240

*Val* valence, *Aro* arousal, *Dom* dominance.

In Fig. 3b and c, sound ratings from JPN present more outliers than the other countries. Table 1 shows descriptive statistics of sounds ratings per country. Analyzing maximum and minimum ratings per emotional dimension in IADS-2, it was observed that countries had different sounds with lowest valence (USA: sound no. 278 “ChildAbuse”. POR: sound no. 285 “Attack2”. MEX: sound no. 279 “Attack1”. JPN: sound no. 255 “ Vomit”). USA and MEX shared the same high valence sound (sound no. 815 “RockNRoll). For POR, it was sound no. 110 “Baby”, and for JPN sound no. 811 “Bach”. Regarding arousal, USA, MEX, and JPN shared the same low arousal stimulus (sound no. 262 “Yawn”). For POR, it was sound no. 151 “Robin”. USA and MEX shared the same sound with the highest arousal rating (sound no. 275 “Scream” ). For JPN it was sound no. 815 “RockNroll” and for POR, sound no. 285 “Attack2”. Regarding dominance, countries had different sounds with lowest rating (USA: sound no. 424 “CarWreck”. POR: sound no. 277 “FemScream3”. MEX: sound no. 285 “Attack2”. JPN: sound no. 600 “BikeWreck”). USA and MEX shared the same sound with highest dominance (sound no. 815 “RockNRoll”). For POR, it was sound no. 100 “Baby”, and for JPN, sound no. 726 “CorkPour”.

MEX and USA presented the highest correlation coefficient in valence (r = 0.949, *p* < 0.001) and arousal (r = 0.893, *p* < 0.001). POR and USA had the highest coefficient in dominance (r = 0.918, *p* < 0.001). JPN presented the lowest correlation coefficient in valence (JPN - USA: r = 0.805, *p* < 0.001), arousal (JPN–POR: r = 0.571, *p* < 0.001) and dominance (JPN–MEX: r = 0.666, *p* < 0.001). In the remaining comparisons, all coefficients were above 0.7 in valence (USA–POR: r = 0.928, POR–MEX: r = 0.932, MEX–JPN: r = 0.858. *P* < 0.001 for all cases), arousal (USA–POR: r = 0.799, USA–JPN: r = 0.760. POR–MEX: r = 0.782, MEX–JPN: r = 0.775. *P* < 0.001 for all cases) and dominance (USA–MEX: r = 0.914, USA–JPN: r = 0.709, POR–MEX: r = 0.900, POR–JPN: r = 0.750. *P* < 0.001 for all cases).

MANOVA showed significant differences in dominance (F = 2.66, *p* < 0.05) but not for valence (F = 2.53, *p* = 0.056) and arousal (F = 0.68, *p* = 0.564). Still, multiple comparison failed to reject the hypothesis of the means of countries being equal (MEX–JPN: T = − 0.58, *p* = 1.000. POR–JPN: T = − 1.78, *p* = 0.456. USA–JPN: T = − 2.55, *p* = 0.065. POR–MEX: T = -1.20, *p* = 1.000. USA–MEX: T = − 1.98, *p* = 0.289. USA–POR: T = -0.78, *p* = 1.000).

For regression analysis, two cases were considered. Since JPN had the lowest correlation coefficients in every emotional dimension, regression models were trained and the best two models were selected: one including ratings from every country (RMSE = 0.453. $R^2$ = 0.850. MSE = 0.205. MAE = 0.3624. Model: Gaussian Process Regression Matern 5/2), and another model excluding ratings from JPN (RMSE = 0.323. $R^2$ = 0.940. MSE = 0.104. MAE = 0.256. Model: Gaussian Process Exponential GPR). From results, it can be observed that the model improved by using only the ratings from USA, POR and MEX, which had the higher correlation coefficients.

### Sex

**Figure 4:**
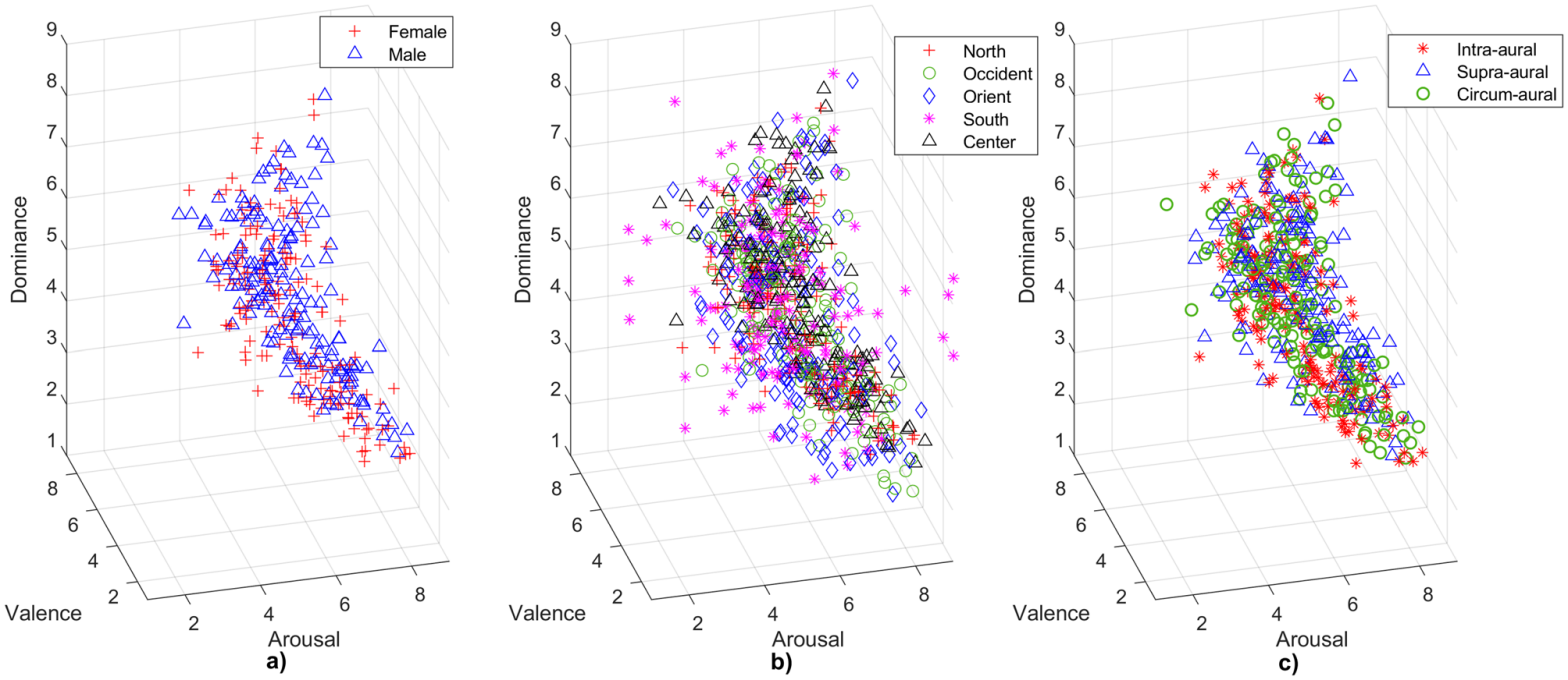
Distribution of mean ratings in the VAD model for MEX. Each plot presents 167 data points representing the mean rating of participants in SAM for each IADS-2 sound. The marker type indicates the group in each category. The 329 ratings were divided within each category as follows: (**a**) Sex group (N: female = 143, male = 186). (**b**) Regions (N: north = 115, occident = 41, orient = 36, south = 13, center = 124). (**c**) Type of headphones (N: intra-aural = 143, supra-aural = 54, circum-aural = 132).

The following number of ratings indicate the participants from each sex group considered to calculate the mean rating of the 167 sounds: (1) female = 143; and male = 186. Figure 4a shows the mean ratings per sex group from the Mexican sample. Data from both sex groups present the same behaviour, showing the same “boomerang” shaped curve as in Fig. 3a. The female group had the lowest (sound no. 279 “Attack1”) and greatest (sound no. 815 “RockNRoll”) mean ratings in valence dimension. For arousal, the male group had the highest (sound no. 275 “Scream”) and lowest (sound no. 262 “Yawn”) ratings. For dominance, the female group had the lowest (sound no. 281 “Attack3”) value, and male group the highest (sound no. 815 “RockNRoll”). Descriptive statistics on the mean ratings are presented in Table 2. Nevertheless, correlation coefficients indicated a positive association between groups (Valence: r = 0.966. Arousal: r = 0.897. Dominance: r = 0.910. *P* < 0.001 for all cases). MANOVA test indicated significant differences in dominance (F = 5.32, *p* < 0.05), confirmed by multiple comparison (T = 2.31, *p* < 0.05). Valence (F = 1.78, *p* = 0.183) and arousal (F = 0.57, *p* = 0.453) did not show significant differences between sex groups.

**Table 2:** Descriptive statistics for each dimension and category according to MEX.

		Val				Aro				Dom			
		M	SD	Min	Max	M	SD	Min	Max	M	SD	Min	Max
**Sex**	Female	4.726	1.848	1.333	8.318	5.824	0.960	3.341	8.067	4.828	1.008	2.805	7.568
	Male	4.981	1.639	1.656	8.095	5.902	0.955	3.091	8.109	5.073	0.932	2.962	7.698
**Region**	North	4.874	1.784	1.500	8.143	5.763	0.996	2.842	8.132	4.905	0.906	3.000	7.457
	Occident	4.771	1.693	1.273	8.100	5.966	1.007	3.263	8.059	4.838	1.098	2.182	7.200
	Orient	4.833	1.826	1.286	8.286	5.782	1.064	3.000	8.571	4.825	1.170	2.214	7.857
	South	5.091	1.866	1.000	9.000	5.607	1.316	1.500	9.000	4.987	1.184	2.000	8.000
	Center	4.906	1.745	1.345	8.345	6.001	1.022	2.828	8.345	5.109	1.045	2.773	7.800
**Headphones**	Intra-aural	4.772	1.794	1.357	8.175	5.815	0.927	3.174	8.236	4.864	1.005	2.717	7.650
	Supra-aural	4.976	1.632	1.850	8.294	5.926	0.989	3.421	8.118	5.171	0.935	2.813	7.941
	Circum-aural	4.941	1.706	1.537	8.286	5.896	0.994	3.095	8.189	4.984	0.998	2.921	7.540

### Regions

The following number of ratings indicate the participants from each region considered to calculate the mean rating of the 167 sounds: (1) north = 115; (2) occident = 41; (3) orient = 36; (4) south = 13; and (5) center = 124. Figure 4b shows the mean ratings of Mexican participants divided in regions. Most data outliers correspond to the southern region. The south had the lowest and highest ratings in valence (min: sound no. 275 “Scream”, sound no. 277 “FemScream3”, sound no. 290 “Fight1”, sound no. 296 “WomenCrying”; max: sound no. 367 “Casino2”, sound no. 728 “Paper1”, sound no. 813 “Wedding”), arousal (min: sound no. 423 “Injury”; max: sound no. 275 “Scream”, sound no. 277 “FemScream3”, sound no. 285 “Attack2”, sound no. 290 “Fight1”, sound no. 310 “Crowd1”) and dominance (min: sound no 241 “MaleCough”; max: sound no. 802 “NativeSong”; sound no. 815 “RockNRoll”). Some sounds had the same greatest or lowest mean ratings in emotional dimensions. Descriptive statistics are presented in Table 2.

Correlation analysis showed that north and center regions had the greatest correlation coefficient in valence (r = 0.969, *p* < 0.05) and dominance (r = 0.893, *p* < 0.05). Occident and center regions had the greatest positive correlation in arousal (r = 0.902, *p* < 0.05). The lowest correlation was observed between occident and south in valence (r = 0.766, *p* < 0.05), arousal (r = 0.463, *p* < 0.05), and between orient and south in dominance (r = 0.274, *p* < 0.05). Coefficients were above 0.5 for all remaining regions in valence (North–occident: r = 0.951. North–orient: r = 0.943. North - south: r = 0.788. Occident–orient: r = 0.946. Occident–center: r = 0.955. Orient–south: r = 0.787. Orient–center: r = 0.955. South–center: r = 0.787. *P* < 0.001 for all cases) and arousal (North–occident: r = 0.835. North–orient: r = 0.751. North - south: r = 0.552. North–center: r = 0.897. Occident–orient: r = 0.675. Orient–south: r = 0.502. Orient–center: r = 0.724. South - center: r = 0.501. *P* < 0.001 for all cases). In dominance, correlation coefficients between some regions were below 0.5 (North - occident: r = 0.843. North–orient: r = 0.814. North–south: r = 0.336. Occident–orient: r = 0.806. Occident–south: r = 0.278. Occident–center: r = 0.867. Orient–center: r = 0.853. South - center: r = 0.321. *P* < 0.001 for all cases).

MANOVA test indicated significant differences in arousal (F = 3.67, *p* < 0.01) but not in valence (F = 0.76, *p* = 0.550) and dominance (F = 1.96, *p* = 0.098). Multiple comparison showed evidence of statistical differences between south–center (T = − 3.31, *p* < 0.05), and south–occident (T = − 3.01, *p* < 0.05) regions, but not between the rest of the regions (North–center: T = − 2.00, *p* = 0.457. Occident–center: T = − 0.30, *p* = 1.000. Orient–center: T = − 1.84, *p* = 0.664. Occident–north: T = 1.70, *p* = 0.886. Orient - north: T = 0.16, *p* = 1.000. South–north: T = − 1.31, *p* = 1.000. Orient–occident: T = − 1.54, *p* = 1.000. South–orient: T = − 1.47, 1.000).

### Type of headphones

The following number of ratings indicate the participants from each headphone group considered to calculate the mean rating of the 167 sounds: (1) intra-aural = 143; (2); supra-aural = 54; and (3) circum-aural = 132. Figure 4c shows the mean ratings of Mexican participants divided in headphone type. Most data outliers correspond to the supra-aural group. In Table 2, intra-aural group had the lowest ratings in valence (sound no. 279 “Attack1”) and dominance (sound no. 424 “CarWreck”), and the highest in arousal (sound no. 275 “Scream”). Supra-aural had the highest ratings in valence (sound no. 815 “RockNRoll”, sound no. 820 “FunkMusic”) and dominance (sound no. 815 “RockNRoll”). Finally, circum-aural had the lowest rating in arousal (sound no. 262 “Yawn”). Only supra-aural group had two sounds with the same greatest valence rating.

Correlation coefficients showed that circum-aural and intra-aural groups had the greatest correlation coefficients in valence (r = 0.979, *p* < 0.05), arousal (r = 0.909, *p* < 0.001) and dominance (r = 0.902, *p* < 0.001). Supra-aural and intra-aural had the lowest coefficients in valence (r = 0.950, *p* < 0.001), arousal (r = 0.880, *p* < 0.001) and dominance (r = 0.778, *p* < 0.001). Coefficients were above 0.7 in supra-aural and circum-aural groups in valence (r = 0.962, *p* < 0,001), arousal (r = 0.892, *p* < 0.001), and dominance (r = 0.827, *p* < 0.001).

MANOVA test indicated significant differences in dominance (F = 4.17, *p* < 0.05), but not in valence (F = 0.68, *p* = 0.507) and arousal (F = 0.59, *p* = 0.555). Multiple comparison showed evidence of statistical differences between intra-aural and supra-aural groups (T = 2.86, *p* < 0.05). Significant differences were not observed between intra-aural–circum-aural (T = − 1.11, *p* = 0.802) and supra-aural–circum-aural (T = 1.75, *p* = 0.241).

### Additional sounds

**Figure 5:**
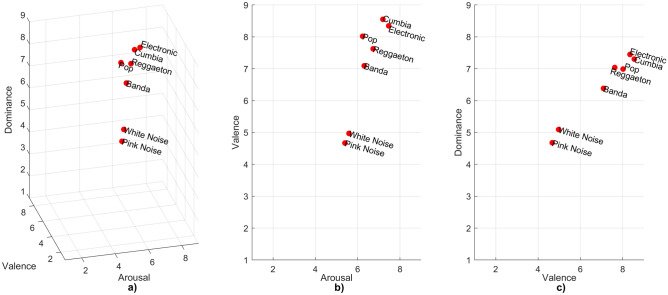
Distribution of mean ratings in the VAD model for seven additional sounds. (**a**) Three-dimensional space. (**b**) Arousal-valence plane. (**c**) Valence-dominance plane. The mean rating of each sound was calculated with the following ratings of participants: (1) Pop = 107; (2) Banda = 107; (3) Electronic = 107; (4) Cumbia = 119; (5) Reggaeton = 104; (6) Pink noise = 106; and (7) White noise = 107.

The following number of ratings indicate the participants considered to calculate the mean rating of the seven additional sounds: (1) Pop = 107; (2) Banda = 107; (3) Electronic = 107; (4) Cumbia = 119; (5) Reggaeton = 104; (6) Pink noise = 106; and (7) White noise = 107. Figure 5 shows the distribution of additional sounds in the three-dimensional space of emotions. Cumbia was the sound with the highest valence (Valence: M = 8.550, SD = 0.938. Arousal: 7.193, SD = 2.154. Dominance: M = 7.303, SD = 2.062), and electronic with highest arousal and dominance (Valence: M = 8.346, SD = 1.166. Arousal: 7.467, SD = 1.717. Dominance: M = 7.449, SD = 1.603). Pink noise had the most neutral sensation across all dimensions (Valence: M = 4.670, SD = 2.032. Arousal: M = 5.396, SD = 2.176. Dominance: M = 4.679, SD = 1.849). White noise had neutral valence (M = 4.972, SD = 2.217) and dominance (M = 5.093, SD = 1.964) but positive arousal (M = 5.589, SD = 2.097). The rest of the music excerpts had positive valence, arousal, and dominance (Pop - Valence: M = 8.019, SD = 1.281. Arousal: 6.234, SD = 2.463. Dominance: M = 6.991, SD = 1.799. Banda–Valence: M = 7.093, SD = 1.851. Arousal: M = 6.308, SD = 1.915. Dominance: M = 6.383, SD = 1.773. Reggaeton–Valence: M = 7.625, SD = 1.553. Arousal: M = 6.731, SD = 1.902. Dominance: M = 7.038, SD = 1.672). None of the sounds caused an aversive effect.

## Discussion

Affective sounds rating are greatly biased by demographic indicators such as gender and nationality^18,30^. The IADS-2 database has been extensively used in emotion research, demonstrating a strong relationship between valence and dominance, indicating that pleasant sounds give the listener a sense of control over emotions, and unpleasant sounds having the opposite effect. Additionally, the sound ratings from many countries present a specific shape in the valence-arousal plane, indicating that pleasant sounds can range between low and high arousal, contrary to unpleasant sounds which show high arousal scores. Results from this work positively correlated to those of USA, POR, and JPN, being the Japanese ratings those who showed a lower correlation to the other countries. Sex differences were observed in dominance, indicating a possible difference in emotion regulation mechanism according to positive and negative sounds. Region analysis demonstrated differences in arousal, which should be confirmed in further studies increasing the sample size of occident and south. Moreover, headphones and audio quality should be taken into account when conducting emotion research, mostly to reduce variability due to audio quality-related aspects.

Ratings from the Mexican sample correlated positively to those of previous validations. However, ratings from JPN had the lowest coefficients compared to other countries. Regression analysis confirmed the hypothesis that nationality influences affective sound ratings as observed in the regression models. Improvement after removing Japanese ratings indicates that IADS-2 responses had a similar tendency in the American, Mexican, and Portuguese samples, hence, a difference caused by culture and traditions.

Comparing sex differences, the female group had the most extreme values in valence and low dominance, and differed significantly only in dominance compared to the male group as observed in the statistical analysis. There is evidence stating that responses from both groups are biased by emotional reactivity^31^, the female group having a greater defensive behavior with unpleasant, threatening stimuli, and the male group having greater appetitive motivation with erotic stimuli^32^, responses attributed to sociocultural factors related to customs and traditions, and the expected roles of sex groups in society. From the previous validations of IADS-2, POR^17^ presented evidence of the female group rating aversive stimulus as more unpleasant compared to the male group. Nevertheless, USA^22^ and JPN^23^ did not find significant differences between sex groups. Future validations in other countries could give a better understanding of the effect of sex on sound ratings since conclusions can not be drawn due to the divided findings of the databases presented in this work.

Furthermore, this study included region analysis. The greatest association between ratings was observed between the center and north regions. This result could be explained by the fact that both regions have the two most competitive urban areas: Mexico City and Nuevo Leon^33^, with the highest population density in comparison to the rest of the Mexican states due to the seek of education and job opportunities. The results from the analysis indicate that sounds from IADS-2 could cause the same sensations in population from these regions. However, the greatest rating differences were observed between south and occident. A limitation of this analysis is that both occident and south had the least number of participants as observed in the supplementary Fig. S1. Future studies should aim to gather more ratings for a better comparison between regions, and determine if geography within a country should be taken into account when stimuli are chosen for emotion elicitation.

Moreover, quality of audio devices, specifically headphones, could modify the auditory content of sounds, distorting the message and, therefore, the intended auditory sensation^34^. Due to the COVID-19 pandemic, this study migrated to a remote version, controlling most variables as possible (e.g., audio quality streaming, devices used in the experiment, audio calibration). To observe the effect of headphones, this study included headphone type analysis in three conventional forms: circum-aural, supra-aural, and intra-aural. In general, conventional circum-aural headphones tend to have a better low-frequency response, and conventional supra-aural headphones reduce sound leakage compared to conventional intra-aural^35^. Improved variations of these models having sealed ear plugs, or frequency-tuned cushions have been developed, but conventional models were assumed for this study. As mentioned in type of headphones section, significant differences were observed in dominance between supra-aural and intra-aural groups. Unexpectedly, differences were not observed between circum-aural and intra-aural groups, which could be contradictory since these two models are the extremes concerning ear device placement. It seems that placing headphones directly on top of the ear affect the sense of emotion control. Tactile and emotional sensation have been proved to be connected, specifically when a device is pressing against a human limb^36^, having an increase in the intended emotion, either positive or negative. Nevertheless, a detailed explanation of the results in this study is not possible because other device factors could not be considered in the analysis due to the wide variety of models used (e.g. frequency response, impedance, brand). This limitation could be addressed in presence mode experiments in future research. Still, country results positively correlated to those of previous laboratory-condition experiments as shown in Fig. 3, indicating that remote experiments concerning affective ratings are plausible when effectively reducing nuisance factors, as previously demonstrated with a smaller sample of another version of IADS^37^.

Finally, additional sounds were included in the study to measure the effect of country-specific music preferences and therapeutic sounds. Noise stimuli are extensively used in psychoacoustic and therapeutic research^19^. In this study, pink noise had neutral (close to 5) ratings in all dimensions. Pink noise is mostly used in audio as a referential signal for audio calibration due to its frequency content adjusted to the human hearing by reducing 3 dB each octave, giving the sensation of a “flat” sound, contrary to white noise, which is perceived with higher loudness at higher frequencies^28^. This finding indicate that pink noise could serve as a transition stimulus between emotion elicitation. Music excerpts were chosen according to the most listened genre in Mexico in mass media^29^. On one hand, the genre with highest valence from all sounds, including IADS-2, was cumbia, suggesting that music can elicit higher valence ratings. On the other hand, the sounds with greatest arousal and dominance were those from IADS-2, “Scream” (no. 275) and “RockNRoll” (no. 815), respectively. A limitation on sounds is that the music excerpts and therapeutic noises had a higher sound fidelity than those from the IADS-2. This characteristic is related to how sounds were produced. High-fidelity stimuli are perceived as natural because they have a high resemblance to the physical stimulus. On the contrary, low-fidelity signals are considered unnatural or technological^18^. Music and sounds produced by audio professional affect emotional responses, such as having an increase in arousal^38^. However, the sound with greatest arousal was from IADS-2 database (no. 275 “Scream”). When studying emotional responses in creative industry research, strict audio recording procedures are encouraged to reduce variability produced by the audio quality. For the most part, IADS-2 and additional sounds ratings could serve as a referential point for choosing and designing auditory material for the intended emotional elicitation in subjects^39^.

## Supplementary Information

Supplementary Figure S1.

## Data Availability

Data is availabile in https://doi.org/10.6084/m9.figshare.19806304.v3.
