# Supplementary material for: Mexican validation of the International Affective Digitized Sounds second edition (IADS-2) and additional sounds
Source: Sci Rep. 2022 Dec 17;12:21824. doi: 10.1038/s41598-022-26320-w (PMC9758458; doi:10.1038/s41598-022-26320-w)
Supplement: Supplementary file 1 — Supplementary Figure S1. [file 41598_2022_26320_MOESM1_ESM.pdf]

Supplementary information

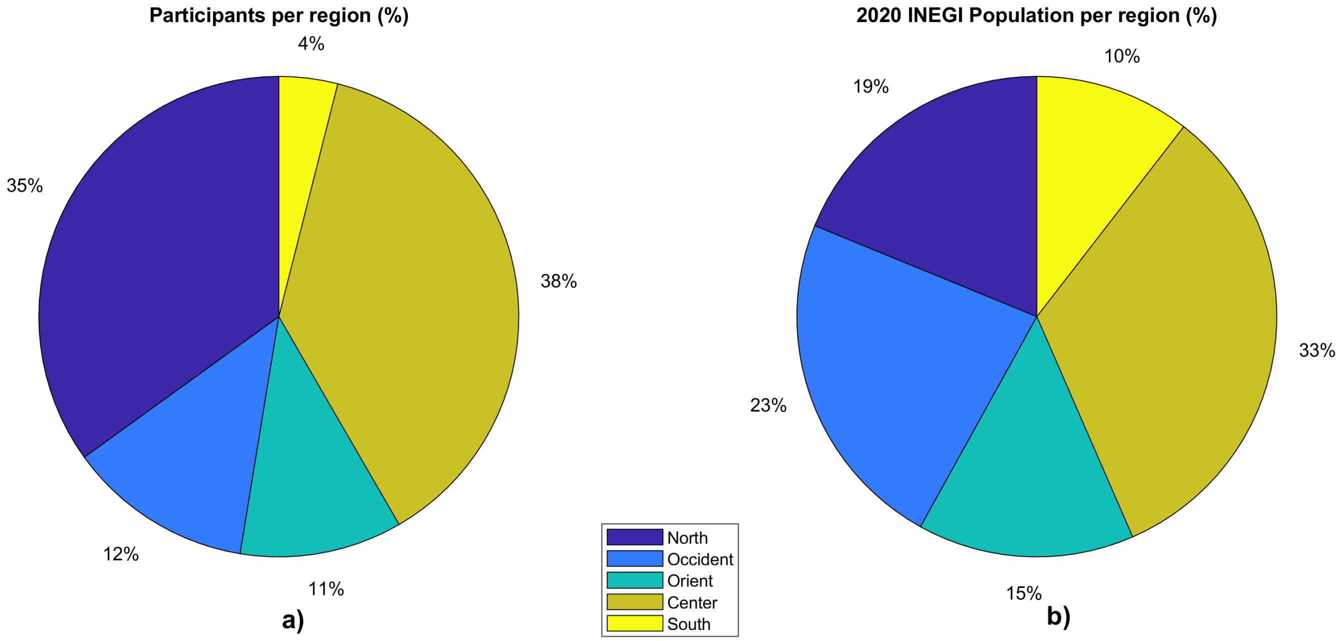

Figure S1. Regional distribution. a) Experiment participants. b) 2020 INEGI Census population. North: Baja California, Baja California Sur, Sonora, Chihuahua, Coahuila, Nuevo Leon and Tamaulipas. Occident: Sinaloa, Durango, Zacatecas, Nayarit, Jalisco, Aguascalientes, Colima, Guanajuato and Michoacan. Orient: San Luis Potosi, Veracruz, Tabasco, Campeche, Yucatan and Quintana Roo. Center: Queretaro, Estado de Mexico, Mexico City, Morelos, Hidalgo, Tlaxcala and Puebla. South: Guerrero, Oaxaca and Chiapas. The INEGI percentage is calculated on a total of 126,014,024 people.
